# Accuracy of cup position following robot-assisted total hip arthroplasty may be associated with surgical approach and pelvic tilt

**DOI:** 10.1038/s41598-021-86849-0

**Published:** 2021-04-07

**Authors:** Shinya Hayashi, Shingo Hashimoto, Yuichi Kuroda, Naoki Nakano, Tomoyuki Matsumoto, Kazunari Ishida, Nao Shibanuma, Tomoyuki Kamenaga, Ryosuke Kuroda

**Affiliations:** 1 Department of Orthopaedic Surgery, Kobe University Graduate School of Medicine, 7-5-1 Kusunoki-cho, Chuo-ku, Kobe, 650-0017 Japan; 2 Department of Orthopaedic Surgery, Kobe Kaisei Hospital, Kobe, Japan

**Keywords:** Medical research, Risk factors

## Abstract

This study aimed to investigate the accuracy of cup placement and determine the predictive risk factors for inaccurate cup positioning in robot-assisted total hip arthroplasty (THA). We retrospectively analyzed 115 patients who underwent robot-assisted THA between August 2018 and November 2019. Acetabular cup alignment and three-dimensional (3D) position were measured using pre- or postoperative computed tomography (CT) data. Absolute differences in cup inclination, anteversion, and 3D position were assessed, and their relation to preoperative factors was evaluated. The average measurement of the absolute differences was 1.8° ± 2.0° (inclination) and 1.9° ± 2.3° (anteversion). The average absolute difference in the 3D cup position was 1.1 ± 1.2 mm (coronal plane) and 0.9 ± 1.0 mm (axial plane). Multivariate analysis revealed that a posterior pelvic tilt [odds ratio (OR, 1.1; 95% confidence interval (CI), 1.00–1.23] and anterior surgical approach (OR, 5.1; 95% CI, 1.69–15.38) were predictive factors for inaccurate cup positioning with robot-assisted THA. This is the first study to demonstrate the predictive risk factors (posterior pelvic tilt and anterior surgical approach) for inaccurate cup position in robot-assisted THA.

## Introduction

Acetabular cup positioning is important for early and long-term outcomes of total hip arthroplasty (THA), and malposition is recognized as a risk factor for postoperative complications, such as impingement, dislocation, and accelerated polyethylene wear, after THA^1–3^. However, even experienced surgeons find it difficult to achieve accurate target cup positioning^4^. Previous reports have demonstrated several factors that lead to inaccurate cup positioning. The factors included patient characteristics comprising body mass index (BMI)^5^, age^6^, gender^6^, and primary diagnosis for THA^7^ or surgery including the performing surgeon's experience^6^, the surgical approach^8^, and the prosthetic components^9^. To resolve this difficulty, various techniques, such as manual guides, intraoperative landmarks, intraoperative X-ray or fluoroscopy, computer navigation, and robotics, were developed to improve the accuracy and precision of acetabular cup positioning in THA^10^. Although computed tomography (CT)-free, fluoroscopy-based, and CT-based navigation systems were developed to improve component positioning in THA^11–13^, several risk factors for inaccurate cup positioning with navigation-assisted THA, such as high BMI and posterior pelvic tilt, persisted^14,15^.

Recently, a new generation of robots was created for assistance during THA and accurate cup positioning^16–18^. Kanawade et al. reported that robot-assisted THA achieved precise cup inclination in 88% and anteversion in 84% within a 10° discrepancy between intraoperative planning angles and postoperative CT validation^18^. Nodzo et al. reported a significant correlation in inclination and anteversion between intraoperative angles and postoperative CT validation^17^.

However, to the best of our knowledge, no study has analyzed the predictive risk factors for inaccurate cup position in robot-assisted THA. This is important because it is difficult to notice an inaccurate cup location and adjust positioning during robot-assisted THA. We hypothesize that robot-assisted THA can achieve accurate cup placement. However, we believe that several predictive risk factors for inaccurate cup positioning may exist. Therefore, this study aimed to investigate the accuracy of cup placement and determine the predictive risk factors for inaccurate cup positioning in robot-assisted THA.

## Patients and methods

### Patients

In this retrospective cohort study, we enrolled 121 consecutive patients (121 joints). Of the 121 joints, 6 were excluded from this study because of missing data sets, including postoperative CT data; finally, 115 patients (115 joints) were analyzed in this study. All patients were diagnosed with osteoarthritis (grade 4 according to the Tönnis classification system) or avascular necrosis of the femoral head (stage 4, 5, or 6 according to the Steinberg staging of avascular necrosis) and underwent robot-assisted THA (MAKO Rio Robot, Ft. Lauderdale, FL) between August 2018 and November 2019. Thirty-one patients had developmental dysplasia of the hip (DDH), which was defined as a lateral center-edge (CE) angle of Wiberg less than 20°^19^. All surgical procedures were performed using a mini-anterolateral approach in the supine position^20^ or posterior approach in the lateral position by three senior surgeons. One surgeon usually used the MIS-anterior lateral supine approach; however, a posterior approach was used when the patient’s BMI was over 35 kg/m^2^ or in severe DDH cases. Conversely, two other surgeons used the posterior approach for all patients.

The demographic data of patients are shown in Table 1.

**Table 1:** Patients background.

Gender	Male 18, female 97
Mean age	67.3 ± 10.6 (y.o.)
Mean BMI	23.5 ± 2.8 (kg/m^2^)
Treated side	Right 66, left 49
Surgical approach	Anterior 29, posterior 86
Mean anterior pelvic tilt angle	1.9° ± 6.2°

### Preoperative plan and surgery

Preoperative CT scans from the level of the iliac wing to the femoral condyle were obtained. The slice thickness was 1 mm, and the CT data were transferred to the MAKO planning module. Subsequently, preoperative planning was performed to determine the optimal component size, angle, and position using the three-dimensional (3D) templating software of the MAKO robotic hip system before surgery. The target cup inclination angle was usually fixed at 40°, and the anteversion angle was determined preoperatively according to Widmer’s combined anteversion theory^21^ with respect to the functional pelvic plane^22^. Pelvic tilt was defined as the inclination angle of the anterior pelvic plane, determined by both the anterior superior iliac spines and the pubic tubercles, relative to the table plane. The patients underwent THA with a Trident hemispherical cup and Accolade II or Exeter v40 stems (Stryker Orthopaedics, Mahwah, NJ, USA).

The robot-assisted THA procedures were performed with the MAKO robotic hip system, which is a robot-assisted computer navigation system that uses the RIO robotic arm (MAKO Rio Robot) to ream the acetabulum and place the acetabular component. After placement of the acetabular cup, one or two screws were inserted, and the intraoperative cup alignment was confirmed by touching five points at the cup edge using the navigation pointer. Full-weight bearing was allowed 1 day after surgery in all patients.

### Postoperative evaluation

**Figure 1:**
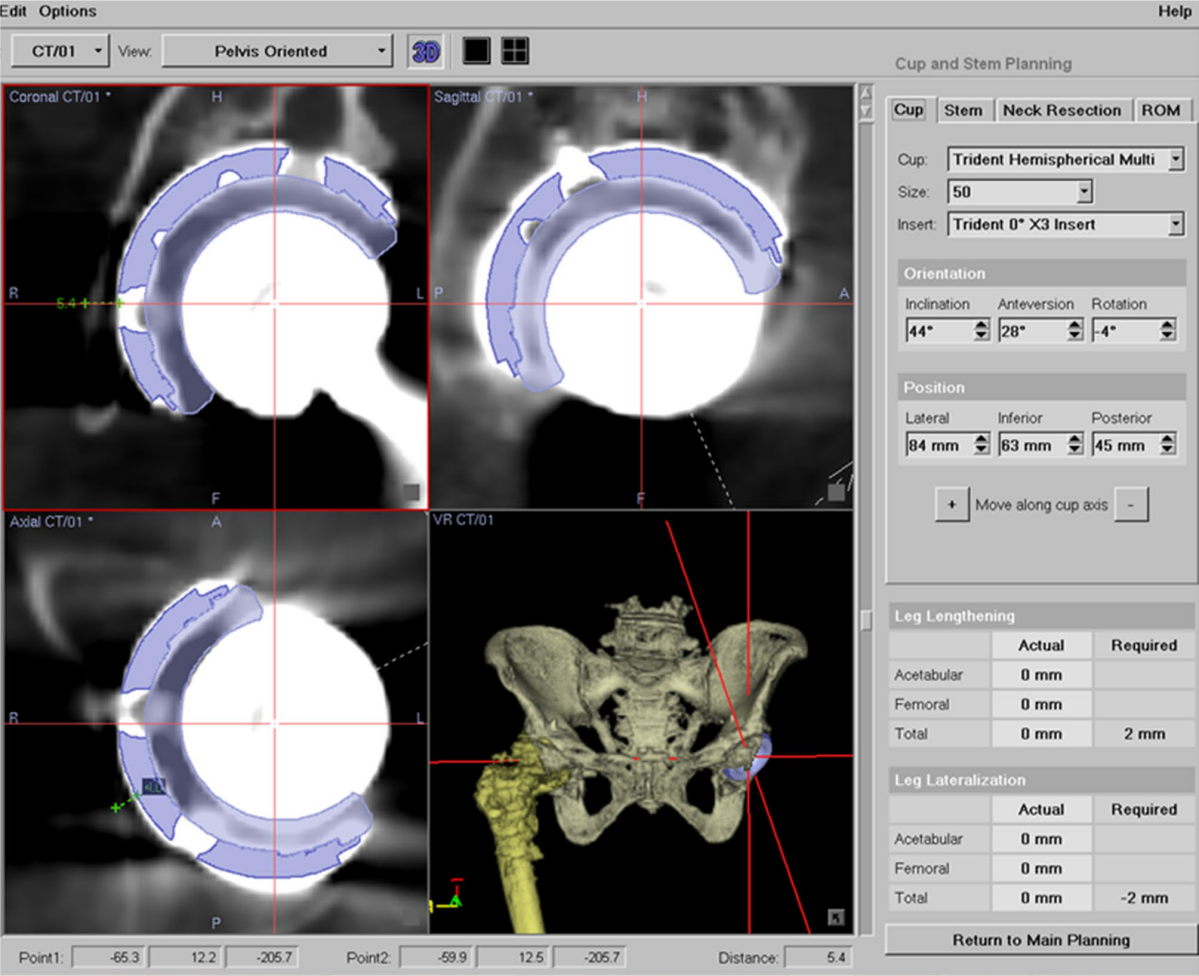
Acetabular cup angles are measured by superimposing the templates of the acetabular cup on postoperative images of the acetabular component.

**Figure 2:**
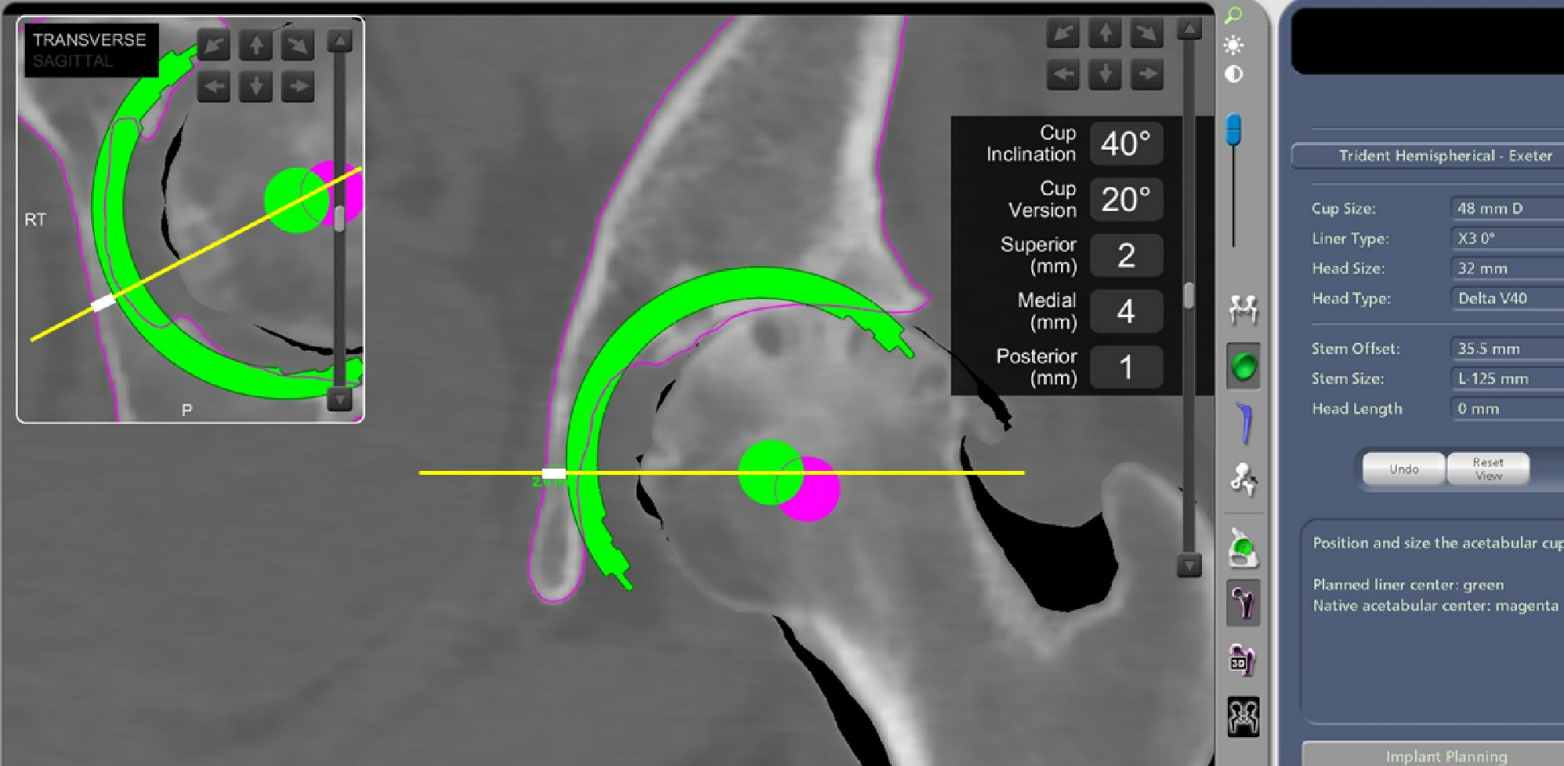
Preoperative cup positions are measured on the MAKO workstation. White lines indicate the distance between the outer edge of the cup and the medial edge of the acetabulum in axial and coronal views.

**Figure 3:**
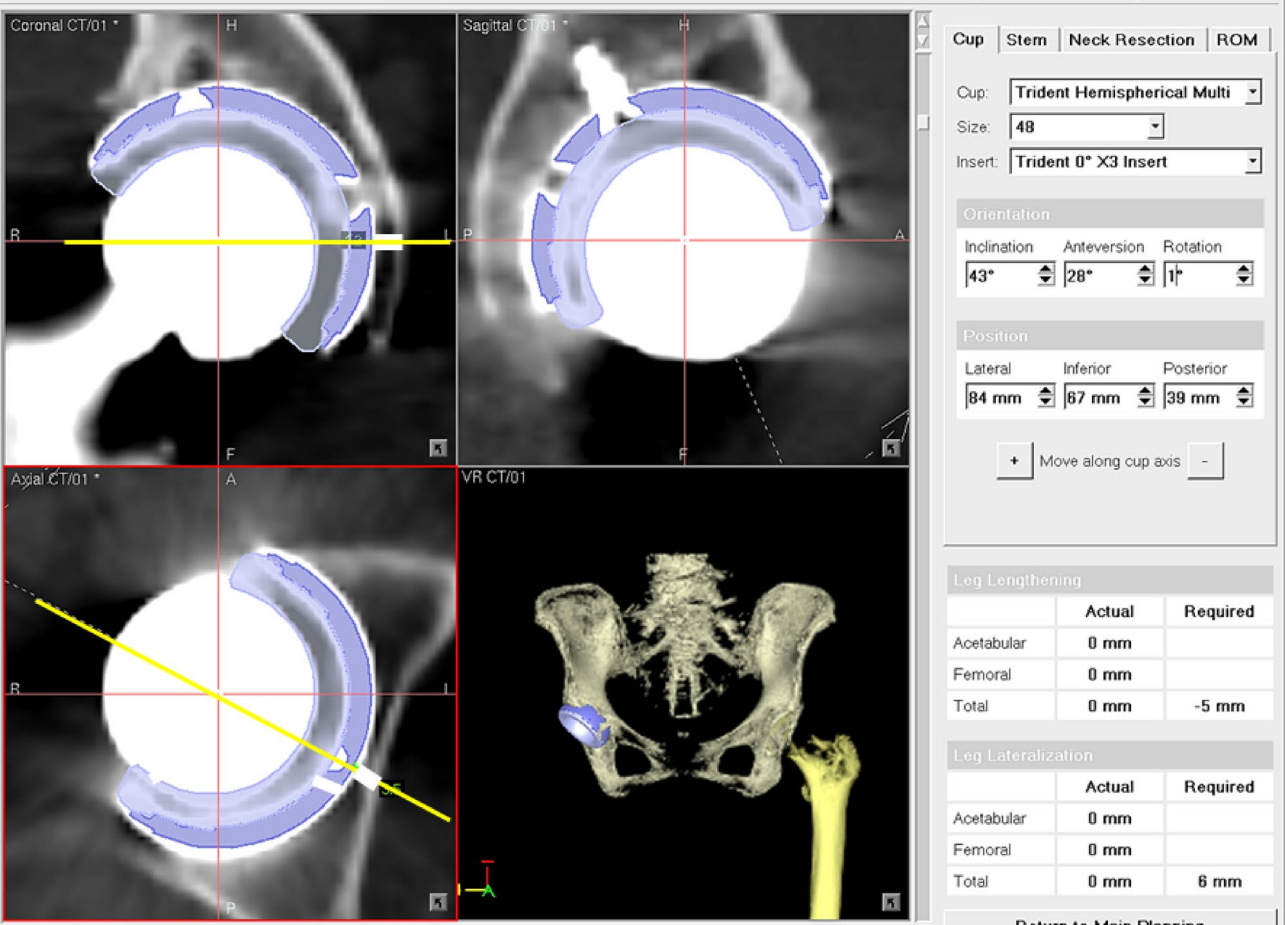
Postoperative cup positions are measured on the OrthoMap 3D Navigation System workstation. White lines indicate the distance between the outer edge of the cup and the medial edge of the acetabulum in axial and coronal views.

Postoperative CT scans were obtained 1 week after surgery, and the data were transferred to the OrthoMap 3D Navigation System (Stryker Orthopaedics). The computer-aided design models of the implants were manually adjusted for postoperative multi-planar reconstruction of the CT images (Fig. 1). Cup inclination and anteversion angles were measured with respect to the functional pelvic plane. To analyze the accuracy of cup alignment, we compared the absolute differences in cup alignment between the postoperative measurements, navigation records, and preoperative plans. To assess the cup position in the axial axis, a normal vector line passing through the cup center was drawn in the axial views of the preoperative plans on the MAKO workstation (Fig. 2) and the postoperative reconstruction image on the OrthoMap 3D workstation (Fig. 3). To assess the cup position in the coronal axis, a horizontal line passing through the cup center was drawn in the coronal views on the preoperative plans and postoperative images (Figs. 2 and 3). The distance between the outer edge of the cup and the medial edge of the acetabulum on the line was measured, and the absolute differences in distance between that assessed in the preoperative plan and the postoperative measurement were calculated in the axial and coronal views (Figs. 2 and 3).

### Statistics

All data are expressed as mean ± standard deviation (SD) unless otherwise indicated. The differences in cup angles between that evaluated in preoperative plans and postoperative CT measurements were analyzed by paired t-test (Table 2). The average measurements of the absolute differences in cup alignment in cases where the posterior approach was used by the surgeons were analyzed using one-way analysis of variance. The outliers of accurate acetabular cup placement were defined as an absolute difference in inclination or anteversion of more than 5° and a difference in distance between preoperative plans and postoperative CT measurements of more than 3 mm, according to previous reports^18,23,24^. To identify predictive factors of the outliers in postoperative cup malposition with robot-assisted THA, outliers and non-outliers were compared using the Fisher’s exact test for nominal variables (Tables 3 and 5) and the unpaired t-test for continuous variables (Tables 4 and 6). Additionally, we performed a multivariate analysis to test the association between acetabular shape, surgical approach, and pelvic tilt with outliers of cup placement (Table 7). Odds ratios (ORs) and 95% confidence intervals (95% CIs) were calculated using the Fisher’s exact test and multivariate analysis. The database was analyzed using SPSS version 16.0 (IBM Corp., Armonk, NY, USA), and p-values < 0.05 were considered statistically significant.

**Table 2:** Results of average cup angles.

	Radiographic inclination (°)	Radiographic anteversion (°)
Preoperative plan	40	19.2 ± 1.9
Navigation record	40.1 ± 2.0	18.8 ± 2.5
Postoperative measurement	39.6 ± 2.6	18.7 ± 3.1
p-value (pre-plan vs post-measure)	0.937	0.140
Absolute difference (postoperative CT-preoperative plan)	1.8 ± 2.0	1.9 ± 2.3

For Fisher’s exact test, a power calculation using G*Power 3^25^ determined that a minimum total sample size of 114 patients would be sufficient to ascertain whether there was a significant difference. A power of 0.8, prespecified significance level of *α* < 0.05, and allocation ratio (N1/N2) based on the preliminary study with 30 cases (with an OR of 5 or more) was set as a clinically meaningful difference. For the unpaired t-test, we calculated the effect size using means and SDs based on the Hedges’ g for each parameter and a 95% CI for effect sizes^26^.

### Ethics

This study complies with the Declaration of Helsinki, study protocols were approved by the ethics committee of the Kobe University Graduate School of Medicine, and all participants provided informed consent for participation.

## Results

### Robot-assisted THA accurately reproduced the preoperative plan of cup angles and positions

Table 2 provides the average angles of cup inclination and anteversion documented in the preoperative plans, intra-operative navigation records, and postoperative measurements. The average inclination angle in the preoperative plan was 40°, and the average anteversion angle was 19.2°. The average angles did not reveal any remarkable change between the preoperative plans and postoperative measurements (Table 2). The average measurements of absolute differences in cup alignment (postoperative CT measurement-preoperative plan) were 1.8° ± 2.0° (inclination) and 1.9° ± 2.3° (anteversion) for all cases (Table 2). Additionally, we compared the average measurements of the absolute differences in cup alignment in cases where the posterior approach was used, and no significant difference in cup alignment was observed among the three surgeons (p = 0.518).

### Reproducibility of cup angles using robot-assisted THA with changes in the pelvic tilt

No notable differences were observed in the frequencies of cup inclination or anteversion outliers on treated sides, surgical approaches, and acetabular shape (Table 3).

**Table 3:** Results of cup angles accuracy.

	Frequency of absolute difference of inclination ≥5° (postoperative CT-preoperative plan)	Frequency of absolute difference of anteversion 5° (postoperative CT-preoperative plan)
**Treated side**		
Right	9.1% (6/66)	4.5% (3/66)
Left	10.2% (5/49)	16.3% (8/49)
p-value	0.592	0.052
Odds (95% CI)	0.88 (0.25–3.07)	0.24 (0.06–1.00)
**Surgical approach**		
Anterior	10.3% (3/29)	17.2% (5/29)
Posterior	9.3% (8/86)	7.0% (6/86)
p-value	0.559	0.141
Odds (95% CI)	1.13 (0.28–4.56)	2.78 (0.78–9.91)
**Acetabular shape**		
DDH	12.9% (4/31)	16.1% (5/31)
Non-DDH	8.3% (7/84)	7.1% (6/84)
p-value	0.484	0.284
Odds (95% CI)	1.63 (0.44–6.01)	2.50 (0.70–8.88)

Table 4 presents the mean values of BMI, age, and pelvic tilt angles of patients in the outlier and non-outlier groups. The average BMI and age of the patients revealed no significant differences for inclination and anteversion angles. However, the mean pelvic tilt angle was significantly different between the outlier and non-outlier groups for anteversion (p = 0.029) (Table 4).

**Table 4:** Results of cup angles accuracy.

	Inclination					
	Absolute difference of inclination ≥5°	Absolute difference of inclination ˂5°	p-value	Hedges’ g	95% CI	
BMI	23.5 ± 2.8	23.1 ± 2.2	0.681	0.18	− 0.45	0.80
Age	67.1 ± 10.7	68.5 ± 9.7	0.673	− 0.14	− 0.77	0.48
Pelvic tilt	1.96 ± 2.0	1.93 ± 1.9	0.988	0.02	− 0.61	0.64

	Anteversion					
	Absolute difference of anteversion ≥5°	Absolute difference of anteversion ˂5°	p-value	Hedges’ g	95% CI	
BMI	23.4 ± 2.4	22.6 ± 2.2	0.272	0.36	− 0.26	0.98
Age	66.7 ± 10.7	70.1 ± 7.8	0.112	− 0.42	− 1.04	0.20
Pelvic tilt	− 1.91 ± 6.1	2.36 ± 5.7	0.029	− 0.74	− 1.37	− 0.11

### Reproducibility of 3D cup positions using robot-assisted THA with variations in surgical approaches

The accuracy of 3D cup positioning was estimated, and the average absolute differences in the distance (postoperative CT-preoperative plan) were 1.1 ± 1.2 mm (coronal plane) and 0.9 ± 1.0 mm (axial plane). No significant differences were observed in the frequency of 3D cup position outliers on the treated sides and acetabular shapes. However, we observed a significant difference in the frequency of 3D cup position outliers between patients who underwent surgery via an anterior or posterior approach (p-value = 0.004) (Table 5).

**Table 5:** Results of 3D cup position accuracy.

	Frequency of absolute difference of distance ≥ 3 mm on coronal or axial plane (postoperative CT-preoperative plan)
**Treated side**	
Right	16.7% (11/66)
Left	10.2% (5/49)
p-value	0.239
Odds (95% CI)	1.76 (0.57–5.44)
**Surgical approach**	
Anterior	31.0% (9/29)
Posterior	8.1% (7/86)
p-value	0.004
Odds (95% CI)	5.07 (1.69–15.30)
**Acetabular shape**	
DDH	12.9% (4/31)
Non-DDH	14.3% (12/84)
p-value	0.559
Odds (95% CI)	0.89 (0.26–2.99)

Table 6 presents the mean values of BMI, age, and pelvic tilt angles of patients in the outlier and non-outlier groups. The average BMI, age, and pelvic tilt were not significantly different between the outlier and non-outlier groups regarding the 3D cup position.

**Table 6:** Results of 3D cup position accuracy.

	Absolute difference of cup position on coronal or axial plane ≥3 mm (postoperative CT-preoperative plan) (mm)	Absolute difference of cup position on coronal or axial plane ˂ 3 mm (postoperative CT-preoperative plan) (mm)	p-value	Hedges’ g	95% CI	
BMI	23.4 ± 2.5	23.9 ± 2.4	0.811	− 0.21	− 0.74	0.32
Age	67.2 ± 10.7	68.4 ± 10.6	0.265	− 0.11	− 0.64	0.42
Pelvic tilt	2.1 ± 6.1	1.9 ± 6.2	0.597	0.03	− 0.50	0.56

### Predictive factors for outliers of postoperative cup placement with robot-assisted THA

We noted that two predictive factors (surgical approach and pelvic tilt) were related to postoperative cup malposition with robot-assisted THA based on unpaired t-test results for continuous variables and Fisher’s exact test results for nominal observations. However, the predictive factors for outliers could be dependent on multiple confounders. Therefore, these two significant predictive factors were used as covariates for multivariate analysis. We discerned that outliers of postoperative cup anteversion with robot-assisted THA were significantly associated with posterior pelvic tilt (OR, 1.1; 95% CI, 1.00–1.23), and the outliers of cup position were associated with an anterior approach (OR, 5.1; 95% CI, 1.69–15.38) (Table 7).

**Table 7:** Results of the multivariate analysis for preoperative factors for inaccurate cup positioning of robotic-assisted THA.

	Preoperative factor	Odds ratio (95% CI)	p-value
Anteversion	Posterior pelvic tilt	1.1 (1.00–1.23)	0.035
Cup position	Anterior approach	5.1 (1.69–15.38)	0.012

## Discussion

We examined the reproducibility of cup placement with robot-assisted THA in this study. This is the first study to demonstrate that a posterior pelvic tilt and anterior surgical approach were significantly associated with postoperative inaccurate cup positioning.

Several reports have analyzed the various factors associated with inaccurate cup positioning with robot-assisted THA^23,27^. Gupta et al. conducted a study in obese patients and found no significant correlation between BMI and acetabular anteversion/ inclination^27^. Their results support our findings that BMI did not affect the accuracy of cup inclination, anteversion, or 3D cup position with robot-assisted THA.

Previous studies have demonstrated a higher complication rate comprising loosening of the acetabular component and postoperative dislocation after THA in DDH patients^28–30^. The reason for the higher complication rate is inaccurate acetabular cup positioning due to an inadequate acetabular roof, a double acetabular floor, the presence of osteophytes, and a difficulty in identifying the accurate orientation of the acetabulum^28–30^. However, several studies described an accurate cup placement with CT-based navigation in DDH cases^12,31,32^. Similarly, we demonstrated no differences in the accuracy of cup positioning between DDH and non-DDH patients after robot-assisted THA.

**Figure 4:**
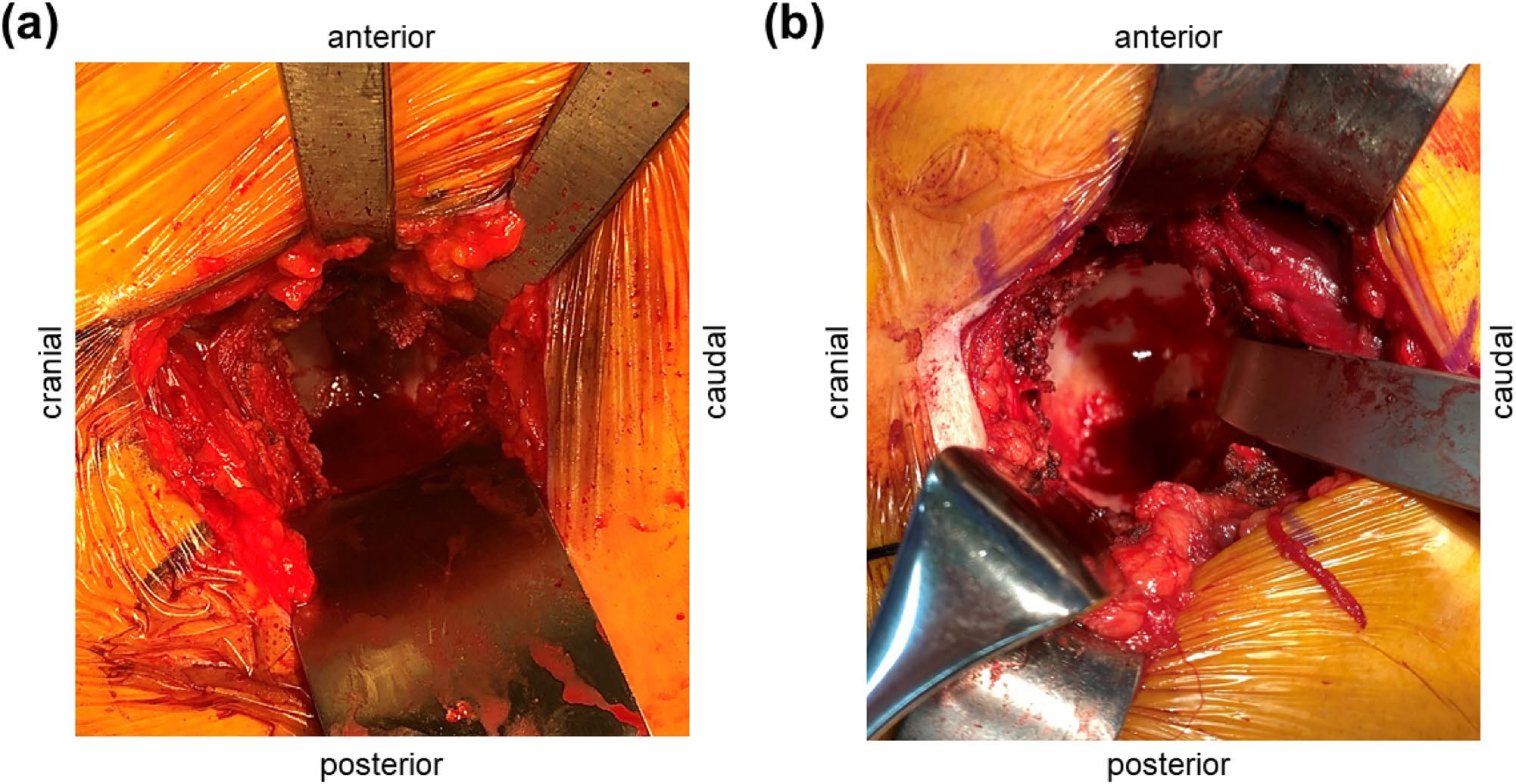
Photographs of the acetabular visual field during registration. **(a)** Mini-anterolateral approach, **(b)** posterior approach.

Several reports demonstrated that the posterior approach could achieve accurate 3D cup positions with navigation THA^33,34^. Nakahara et al. demonstrated in a case series using the posterior approach that the CT-based navigation accuracy of the implant position, which was defined as differences between the postoperative measurements on CT images and intraoperative records on the navigation system, was within a 2-mm difference^34^. However, we did not find any literature focused on 3D cup positions of the mini-anterior surgical approach with robot-assisted THA. In this study, we observed that a mini-anterior approach affected 3D implant position with robot-assisted THA. Minimally invasive surgical approaches are currently used for THA. Mini-incision THA reduces postoperative pain and blood loss, leads to a speedy recovery, and reduces hospital stay compared to THA performed using a standard approach^35^. However, some researchers are concerned that mini-incision THA may introduce new potential problems related to a reduced visual field during surgery, such as implant malposition, neurovascular injury, and poor implant fixation^36^. The visual field during manual registration of landmarks during a mini-anterior approach in the supine position was much smaller than that during a posterior approach, with difficulty in the registration of the anterior and posterior wall edges (Fig. 4). A sufficient visualization of the surgical field is essential for the registration of the cup positioning, and the idea of improving accuracy despite the approach is needed for robot-assisted THA implantation.

We also determined that a posterior pelvic tilt affected the accuracy of postoperative cup anteversion with robot-assisted THA. Previous simulation studies suggest that changes in pelvic tilt would lead to changes in the acetabular anteversion^37^. Yamada et al. reported that the accuracy of cup anteversion in CT-based navigation was lower in patients with a greater posterior pelvic tilt^15^. Hasegawa et al. also stated that posterior pelvic tilt affected the accuracy of postoperative cup anteversion angle in a mini-anterolateral approach using an accelerometer-based portable navigation system^38^. We had previously reported that preoperative posterior pelvic tilt was significantly associated with greater pelvic motion on the axial axis during surgery^39^, and the greater motion could cause anteversion errors even with robot-assisted THA.

Our study had some limitations. First, the number of patients included in the study was too small to analyze all the aspects of robot-assisted THA. Secondly, this was not a randomized trial but a retrospective cohort study with inherent limitations.

## Conclusion

This is the first study to determine the predictive risk factors of inaccurate cup position in robot-assisted THA. Posterior pelvic tilt and anterior surgical approach were significantly associated with postoperative inaccurate cup positioning in robot-assisted THA. Thus, it is imperative to pay attention to these findings in THA cases to reduce errors.
